# Care for women in the pregnancy-puerperium cycle from the perspective of health professionals in the light of complexity thinking*

**DOI:** 10.1590/1518-8345.7313.4458

**Published:** 2025-02-03

**Authors:** Dirce Stein Backes, Taina Ribas de Morais, Caroline Brondani Rosa, Leris Salete Bonfanti Haeffner, Dulce Maria Pereira Garcia Galvão, Adriana Dall’Asta Pereira

**Affiliations:** 1 Universidade Franciscana, Santa Maria, RS, Brazil; 2 Scholarship holder at the Conselho Nacional de Desenvolvimento Científico e Tecnológico (CNPq), Brazil; 3 Scholarship holder at the Coordenação de Aperfeiçoamento de Pessoal de Nível Superior (CAPES), Brazil; 4 Escola Superior de Enfermagem, Coimbra, Portugal

**Keywords:** Nursing, Comprehensive Health Care, Pregnancy, Postpartum Period, Primary Health Care, Qualitative Research

## Abstract

to understand the meaning of care for women in the pregnancy-puerperium cycle from the perspective of health professionals in the light of complexity thinking.

qualitative research anchored in the theoretical assumptions of complexity thinking: inseparability, complementarity and interconnectedness. Twenty-three professionals in the field of maternal and child health from the southern region of Brazil took part. The data was collected through individual interviews and analyzed using the thematic analysis technique.

the three themes of analysis were the inseparability of care for pregnant women and puerperal women in the complexity of being a woman and a mother; the complementarity of care for women in the pregnancy-puerperal cycle; the interconnectedness of professional knowledge and practices.

the meaning of care for women in the pregnancy-puerperium cycle, as understood by professionals, is associated with an integral understanding of the woman - pregnant woman, puerperal woman, mother, as an indivisible unit.

## Highlights

**(1)** Pregnant and postpartum women are conceived in the indivisible complexity of being a woman and a mother

**(2)** It suggests overcoming reductionist and simplifying approaches.

**(3)** It signals interprofessional ruptures and advances.

**(4)** It indicates multi-professional movements in maternal and child health.

**(5)** It supports complexity thinking as a relevant and current reference.

## Introduction

The pregnancy-puerperium cycle is a journey in a woman’s life that requires unique and multidimensional care, par excellence. Although transitory, this period is triggered by continuous and definitive changes in the dynamics and organization of being a woman/mother, both in the physical and emotional dimensions and in the socio-cultural dimensions, which transcend maternity^(2022-2018)^.

This period involves an indivisible dynamic that requires health professionals to understand and provide comprehensive care to pregnant women in the physical, emotional, social and spiritual dimensions. However, professional care is often limited to the physical dimension and its contours with illness, the risk of complications and specific interventions associated with identifying and preventing illnesses^(2021-2022)^.

Studies show that there is little mention of the importance of managing the emotional, social and spiritual demands of pregnant and puerperal women, due to the dichotomous and fragmented approaches to the pregnancy-puerperal cycle. On the other hand, they recognize that comprehensive, continuous and complementary care is capable of minimizing emotional and physical tensions and improving the well-being of both mother and child^(2023-2020)^. Social support and emotional attention favor the mother-baby bond and spirituality plays an essential role in reducing stress, as well as providing coping mechanisms^(2021)^.

Expanded, multi-professional care for women in the pregnancy-puerperal cycle can therefore stimulate interactive, complementary and systemic processes capable of involving the different areas of health professional knowledge. This requires references that broaden thinking and conceive of the woman, pregnant woman, puerperal woman and mother as a complex unit, not reduced to dissociable phases or dimensions^(2020-2018)^.

The theoretical assumptions of complexity thinking in this study include inseparability, complementarity and interconnectivity. These pillars transcend reductionist Cartesian thinking governed by order, separability and predictability, which has been shaken by the evolutionary development of the sciences. By understanding the pregnancy cycle in its uniqueness and multidimensionality, complexity thinking tends to overcome hierarchical approaches to knowledge, recover the relationships that exist between knowledge and enable broader, multidimensional care for women in the pregnancy-puerperal cycle^(2015)^.

In the search for a broader, multi-professional understanding of what it means to be a woman/mother, beyond the pregnancy-puerperal period, and in the desire to contribute to Nursing’s own knowledge in the prospect of thinking that integrates, distinguishes and complements, the research question is: what is the meaning of singular, multidimensional care for women in the pregnancy-puerperal cycle for health professionals? Based on the above, the aim is to understand the meaning of care for women in the pregnancy-puerperium cycle from the perspective of health professionals in the light of complexity thinking.

## Method

### Type of study

Qualitative research anchored in the theoretical assumptions of complexity thinking, such as: inseparability, complementarity and interconnectivity. Complex thinking enables a methodological path in which the researcher is induced to learn, invent and (re)create themselves along the way, through interpretative processes that induce advances in the field of health management and care. As a method, complexity thinking broadens the dynamic understanding of being a woman (pregnant woman, puerperal woman, mother), permeated by an organization and singular experiences woven together, in which assistance/care needs to consider the inseparable unity in the different phases, as well as each phase - pregnancy, childbirth, puerperium - in the complex whole^(2015-2014)^.

### Scenario, participants and selection criteria

Twenty-three professionals working in Primary Care Center, specifically in the area of maternal and child health, participated in the study. All professionals who had worked in the area of maternal and child health in the central region of Rio Grande do Sul for more than two years were invited to participate by means of a formal individual invitation. However, professionals with proven experience in providing care to women during pregnancy and childbirth were included. The exclusion criteria were: health professionals who worked only during pregnancy or the puerperium, and professionals who were absent from work for some justified reason.

Initially, a total of 33 participants with significant regional representation in the area of maternal and child health were estimated, but only 23 health professionals met the inclusion and exclusion criteria, namely: 14 nurses, six physicians and three dentists. Participants were contacted by email, using the contact information provided by the regional health coordinator. Data were collected at each participant’s place of work, in a space reserved and indicated by the professional.

### Data collection technique and period

Data were collected through individual interviews between March and August 2022 by a nurse previously trained for this purpose. The interviews were conducted based on the following questions: What does expanded, multidisciplinary care for women during pregnancy and childbirth mean to you? In your opinion, how can we promote complementary and integrative care for women during pregnancy and childbirth? How can we advance and enable more interconnected and horizontal practices for women during pregnancy and childbirth? These questions were further explored in order to contemplate new meanings and perceptions.

The individual interviews were conducted by only one interviewer, at a place, day, and time indicated by the study participants. The focus was on a welcoming and noise-free environment to ensure the uniqueness and privacy of each participant. The interviews were recorded on a digital recorder and lasted approximately 40 minutes. Once organized, the interviews were transcribed in full using a text editor by two researchers. The transcribed data set resulted in 68 pages, which were approved by the interviewees.

### Data processing and analysis

The data were analyzed based on the thematic analysis technique^(2020)^, systematized in six stages, as follows: a) Setting, a phase in which the researchers immersed themselves deeply in the data, in order to become broadly familiar with the textual elements; b) Inductive compilation of initial codes, a stage in which the researchers manually and systematically coded the data set with full and equal attention to each textual element; c) Generation of themes of meaning - a stage in which the different codes were combined into comprehensive themes; d) Review of themes - a stage in which the researchers refined the themes, in order to denote, separately, the proximities and distinctions between the themes; e) Designation of themes - a stage in which the researchers identified the essence/centrality of each theme, in particular and in the set of themes; f) Preparation of the research report - a stage in which the researchers refined, in detail, the data set and, finally, wrote the report. This analysis process was not, however, punctual and linear, but demanded inductive and recursive movements, as proposed by complexity thinking.

The data analysis process began when the first interviews were conducted, based on patterns of meaning. To this end, ideas, insights, drafts and schemes were constantly recorded, not with the aim of achieving accuracy, but with the aim of achieving depth and meaning, in light of complexity thinking.

### Ethical aspects

In order to comply with ethical issues, the recommendations of Resolution No. 466/2012 of the National Health Council were considered. To maintain anonymity, the participants’ statements were identified throughout the text by the letter “HP” (Health Professional), followed by an Arabic numeral according to the order of the statements: HP1... HP23. The research participants were informed about the purposes, the methodology used, the right to free access to the data and to withdraw at any time. Their consent and acceptance to the research was given through the signing of the Free and Informed Consent Form. The project was approved by the research ethics committee under number: 5,183,232 and Certificate of Presentation of Ethical Appreciation (CAAE): 54380521.2.0000.5306.

## Results

Of the 23 participants, the average age was 38 years and the average level of education was higher education. The professionals had worked in the area of maternal and child health within the scope of Primary Health Care for 3 to 19 years.

The following three themes are presented in light of the complexity approach: The inseparability of care for pregnant and postpartum women in the complexity of being a woman and a mother; The complementarity of care for women in the pregnancy-puerperal cycle; The interconnectivity between knowledge and professional practices.

### The inseparability of care for pregnant and postpartum women in the complexity of being a woman and a mother

The participants’ statements highlighted the need to invest in the inseparability of comprehensive care for women, that is, beyond the unique phases of a woman’s life, such as pregnancy, childbirth and the postpartum period. Some participants mentioned that this cycle is sometimes fragmented and conducted in separable parts: *I often feel that each professional perceives and cares for one part and fails to realize that the woman is the same, unique in pregnancy, childbirth, postpartum and motherhood* (HP 5).

The pregnancy-puerperal cycle is considered by participants to be an indivisible period that cannot be reduced to consultations, exams, disease prevention or groups of pregnant women, since the favorable or unfavorable outcome of one of the parts compromises the dynamics of the woman in her vital organization. One participant emphasized, more incisively, that the woman needs to be welcomed and understood in her situational context and that the engagement of all professionals and sectors should lead to comprehensive and complementary care: *Pregnancy, childbirth, and the birth of a baby are unique moments in the life of a woman and her family. Everything needs to work in an integrated and continuous way. Women cannot be assisted in stages, but rather welcomed and understood in all dimensions. The family must be welcomed and integrated throughout this process* (HP 9).

The quality of care for pregnant and postpartum women needs, according to the participants of this study, to evolve and achieve a new meaning, considering that care remains fragmented and organized into distinct and specific phases. In this sense, quality was translated into the professional ability to enable dialogic spaces organized into care networks: *It is clear that we have evolved a lot in the care provided to pregnant and postpartum women, but we still work in isolation and do not see pregnant women, postpartum women, and mothers as unique and part of a network* (HP 10). *Sometimes everything becomes very separate and we forget that the woman who goes through the different points of the network is the same* (HP 12).

Some participants reported some discomfort and clearly recognized the weaknesses present in comprehensive care for women. They realized that quality care consists of evolving theoretical understanding and the practice of welcoming women – pregnant women, postpartum women, mothers – in their unique and multidimensional dimension. In this regard, they referred to concrete spaces for dialogue between professionals from different points in the network, in order to broaden understanding and discuss joint intervention strategies: *Everyone should be aware of the role of each professional in maternal and child health. Sometimes we only worry about our part and forget that the pregnant woman and the postpartum woman should be at the center of care* (HP 15)*. Regarding cesarean sections, we do not have a single language. Everyone explains it in their own way and based on their own vision and understanding. This needs to improve a lot so that women can effectively occupy the center of care* (HP 17).

Although most participants reported advances in care for women during the prenatal period and childbirth, a small group reinforced the need to improve postpartum care, which is generally relegated to a secondary role. One participant’s testimony highlighted the progress made in humanizing childbirth, but the postpartum period has not advanced at the same rate: *Here we do our best to ensure that this woman has a calm and humanized birth, but when I spoke to a postpartum woman, she told me that no one else had contacted her after the birth of the child* (HP 23).

The participants’ speeches generally showed a desire for evolution both in terms of broader theoretical understanding and in comprehensive care for women during pregnancy and childbirth. At times, participants were unable to express themselves scientifically, but they recognized that women users, whether pregnant or postpartum, are unique beings and part of a broader personal, family and social reality.

### Complementarity in assistance to women in the pregnancy-puerperal cycle

Several participants mentioned that, generally, each professional sees and performs only their part, their routine and, consequently, defends their local specificities without considering the health network: *I notice the lack of alignment among professionals working in maternal and child health. Women are the same and the procedures need to converge to provide better care* (HP 4).

One participant, in particular, noted that counter-referrals rarely occur on the part of professionals working in the hospital area. From this perspective, he highlighted the lack of communication and dialogue between the various sectors and actors in the network. *It has happened that a child was born with problems and developed cardiorespiratory arrest and we at the Health Unit were not aware of it. There is a lack of communication and understanding of the work of each professional. Each one thinks they are doing the best, but we do not know what is happening on the other side. Each one defends their part* (HP 13).

The participants’ statements highlighted the importance of the complementary and continuous work process between the various services in the health network. From this perspective, one participant reflected that within the Unified Health System (SUS) no one works alone or in isolation and that the user must be welcomed as unique and part of a network, in which each point is complementary to the other. *We need to understand that nothing happens in isolation or separately in the SUS. Women cannot wander from one service to another. We are all responsible, no matter which unit I work in. We need to look at the SUS as a large system, which only works in cooperation with all professionals in the network* (HP 18).

One participant, in particular, demonstrated that in addition to technical knowledge, professionals need to have broad and in-depth knowledge of how the maternal and child health network works: *You need to understand how the maternal and child health network works as a whole. Our tendency is often to only look at what happens in the workplace itself without realizing the consequences of each professional act on the user’s life. We would need to have more meetings to study and understand what happens at the various points of the network* (HP 19).

Most of the testimonies indicated the need to develop new ways of thinking and acting in the care provided to women during pregnancy and childbirth. Although they recognize initiatives and advances in the area, not all professionals are properly trained and in tune with the principles and guidelines that govern comprehensive care for women’s health.

### The interconnectivity between knowledge and professional practices

Several participants referred to the urgency of promoting and expanding spaces for dialogue to share knowledge and practices among professionals from different points of the maternal and child health network. In the speech of one professional, more specifically, the lack of dialogue between professionals, sectors and services was evident, when mentioning: *I see that such beautiful things happen in the services, but we don’t publicize them and we don’t find out about them. The achievements of a service can boost other initiatives, but sometimes we don’t find out about them. The other day, a user told me about everything the unit promotes to make people feel welcomed* (HP 6).

It was noted in other statements that new scientific evidence in the area is not always considered and applied in professional practice. In this sense, some participants referred to professionals who guide their practice in traditional models, that is, without scientific criteria and evidence. *There are professionals who continue to defend their traditional practices, which today make no sense at all. Many of them are even contraindicated. I see this as a great challenge. Things change and our minds also need to evolve* (HP 14).

Other participants also referred to prospective professional training. They recognize that health professionals are not always trained in the logic of cooperation and interconnectivity between knowledge and practices supported by the best scientific evidence. From this perspective, they noted that overcoming fragmented and dichotomous processes necessarily involves professional training based on references that broaden multiprofessional perspectives. *Vocational training needs to evolve in many areas. Theory does not always have meaning in practice, just as practice without theory has no meaning. How should things evolve? I understand that there needs to be more dialogue and interaction between universities and services* (HP 20).

The necessary dialogue was also demonstrated in multiprofessional and interprofessional actions. For most participants, the quality of care for women during pregnancy and childbirth requires broader perception and multiprofessional actions, based on the induction of shared spaces for knowledge construction and collegial decision-making. *We need to learn from each other. Quality care is not achieved through the work of a single professional area or service. Each professional has their own perception and together we will have greater reach* (HP 21).

Continuing education in health is, in the view of the participants, an important tool for aggregating and promoting more dialogic, collaborative and complementary professional attitudes among professionals who are part of the maternal and child health network. In addition to technical skills and competence, it is essential that health professionals are able to transcend their knowledge and their space of professional practice.

## Discussion

The thought of complexity leads to the joint weaving of the tangle of threads that integrate, complement and dynamize the care provided to pregnant and postpartum women in the indivisible complexity of being a woman and a mother. The different phases and experiences of women are articulated to produce the whole that reflects on the different dimensions^(2015-2014)^. Therefore, complexity thinking is a challenge to knowledge and an impetus for prospective evolution in the area of maternal and child health.

Instead of conceiving and promoting assistance in phases - pregnancy, childbirth, postpartum, motherhood, women need to be understood in their indivisible existential reality, which involves expanded, singular, complementary knowledge and interconnected with facts, experiences and paths that broaden perspectives and strengthen bonds^(2023-2020)^. Expanded and comprehensive care for women during pregnancy and childbirth requires, from this multifactorial and prospective perspective, to include complementary and multidisciplinary approaches and practices, with a view to reaching the physical, psychological, social and spiritual dimensions.

Scientific evidence corroborates this expanded and evolutionary thinking by demonstrating the need to center care on women – a complex unit, regardless of the phase or period in which they find themselves. By encompassing the complexity of care/assistance, professionals recognize women’s right to autonomous decisions and enable complementary and inseparable paths in the pregnancy and childbirth cycle^(2024-2022)^.

Moving towards the theoretical assumptions of complexity thinking is urgent so that, in the face of the challenges related to the hegemonic model of intervention in the area of maternal and child health, a complex vision of the world, of human beings, and of health care from an interprofessional perspective is favored. Overcoming prevalent approaches in the care of women in the pregnancy-puerperal cycle therefore requires a broader and multiprofessional perception, based on shared spaces for the construction of knowledge^(2024)^.

By recognizing that “women cannot be assisted in stages”, it is understood that the whole goes far beyond the simple sum of the parts/periods of the pregnancy-puerperal cycle. Thus, as the parts integrate and complement each other to form the whole, new ideas, behaviors and theoretical-practical developments are produced. Space is given to the sharing of knowledge, the interconnectivity of dialogues and the inseparability of approaches and professional practices^(2023)^.

Technical and scientific advances increasingly depend on knowledge articulated and shared among professionals from different areas of knowledge. In this sense, it is necessary to foster references that transcend traditional and inflexible professional positions still present in health settings, as evidenced in this study: “they continue to defend their traditional practices, which today make no sense at all”. In addition to new policies and strategic actions aimed at pregnant and postpartum women, new individual and collective behaviors and attitudes are increasingly needed, based on shared approaches and supported by the best scientific evidence^(2023-2023)^.

However, how can we uncover new theoretical possibilities and promote professional investments that consider women’s care in its uniqueness and multidimensionality? The promotion of more advanced and complex knowledge and practices in the health area necessarily implies evolving in systemic thinking and recognizing interprofessional and multidimensional approaches capable of transcending the fragmentation and linearity of being and promoting care for women in the pregnancy-puerperal cycle^(2022-2020)^.

Conceiving the quality of care in the pregnancy-puerperal cycle in the indivisible complexity of being a woman and a mother goes back, in the logic of complexity thinking, to a historical-hegemonic tradition, in which verticalized, prescriptive and predictable relationships prevailed, in which the woman’s autonomy and decision-making are often disregarded^(2022)^. Under this traditional hegemonic bias, women were generally deprived of their rights and rarely encouraged to take leading roles and make autonomous decisions^(2023)^. This traditional-reductive thinking and acting does not agree with the theoretical assumptions of inseparability, complementarity and interconnectivity in light of complexity thinking.

Complex phenomena emerge from the dynamic behavior of interacting and associative units referred to as self-organizing, which occur in systems that are dissipative and nonlinear^(2023)^. Under this thinking, knowledge of the whole depends on the parts, just as knowledge of the parts depends on knowledge of the whole, as demonstrated by the participants. This process reflects the professional capacity to interconnect different areas and services that make up the maternal and child health network and, consequently, to dialogue with the diverse professional knowledge.

The human being - woman - is par excellence a complex unit, that is, producer and product of a dynamic, evolutionary and multidimensional complexity^(2021)^. Thus, although the inseparability, complementarity and interconnectivity in the pregnancy-puerperal cycle are denied, comprehensive care for women - pregnant women, puerperal women, mothers - necessarily implies breaking with hegemonic models and reforming the thinking of health professionals. It is not enough to have an organized and articulated network in maternal and child health if, at the same time, the thinking and actions of health professionals remain verticalized, fragmented and based on disciplinary and one-dimensional knowledge.

Evolving in thinking means, from an evolutionary and prospective perspective, understanding women in their uniqueness and complementarity; transcending the disciplinary barriers of the health area and achieving an integrative, collaborative and inseparable thinking^(2015-2014)^. This evolutionary path implies recognizing that the quality of care for women is associated with the capacity to evolve in reflexivity that involves contradictory and apparently antagonistic actions to generate complex and systemic phenomena.

The contributions of this study to the advancement of scientific knowledge in the area of maternal and child health are associated with overcoming a reductive way of thinking that conceives women (pregnant women, puerperal women, mothers) in fragmented and dissociable parts. Comprehensive and quality care for women in their pregnancy-puerperal cycle necessarily implies ruptures and advances, which can be led by nurses imbued with an expanded, integrative and prospective way of thinking. It is essential that nurses empower themselves with this unique knowledge and contribute, prospectively, to an evolutionary performance in the context of maternal and child health.

A limitation of this study is the fact that not all participants were representative of the area and were previously planned and included in the study. Another limitation is associated with the short time participants had to conduct meaningful and in-depth interviews.

## Conclusion

The meaning of care for women during pregnancy and childbirth, in the understanding of professionals, is associated with the integral understanding of women – pregnant women, puerperal women, mothers – as indivisible units. In addition to the pregnancy and childbirth cycle, women need to be understood in their unique and indivisible experiences.

The expanded and contextualized understanding of care/assistance, as a complex unit – singular and multidimensional – is essential for the induction of autonomy, creativity, interactivity, and ultimately close, dialogical and humanized relationships in the context of maternal and child health.

This research highlights the importance and recommends new studies on the necessary evolution of thinking in the care of pregnant and puerperal women, in order to promote integrative and collaborative approaches among professionals and between professionals and health users.
