# Supplementary material for: Care for women in the pregnancy-puerperium cycle from the perspective of health professionals in the light of complexity thinking
Source: Rev Lat Am Enfermagem. 2025 Feb 3;33:e4458. doi: 10.1590/1518-8345.7313.4458 (PMC11789718; doi:10.1590/1518-8345.7313.4458)
Supplement: Translation

# Translation: Asistencia a la mujer en el ciclo gravídico-puerperal desde la perspectiva de los profesionales de la salud a la luz del pensamiento de la complejidad*

**DOI:** 10.1590/1518-8345.7313.4457

**Authors:** Dirce Stein Backes, Taina Ribas de Morais, Caroline Brondani Rosa, Leris Salete Bonfanti Haeffner, Dulce Maria Pereira Garcia Galvão, Adriana Dall’Asta Pereira

**Affiliations:** 1 Universidade Franciscana, Santa Maria, RS, Brasil; 2 Becaria del Conselho Nacional de Desenvolvimento Científico e Tecnológico (CNPq), Brasil; 3 Becaria de la Coordenação de Aperfeiçoamento de Pessoal de Nível Superior (CAPES), Brasil; 4 Escola Superior de Enfermagem, Coimbra, Portugal

**Keywords:** Enfermería, Atención Integral de Salud, Embarazo, Periodo Posparto, Atención Primaria de Salud, Investigación Cualitativa

## Abstract

comprender el significado de la asistencia a la mujer en el ciclo gravídico-puerperal, desde la perspectiva de los profesionales de la salud a la luz del pensamiento de la complejidad.

investigación cualitativa anclada en supuestos teóricos del pensamiento de la complejidad: indisociabilidad, complementariedad e interconectividad. Participaron 23 profesionales del área de salud materno-infantil de la región sur de Brasil. Los datos fueron recolectados mediante entrevistas individuales y analizados mediante la técnica de análisis temático.

las tres temáticas de análisis fueron la indisociabilidad de la asistencia a la gestante y puérpera en la complejidad de ser mujer y madre; la complementariedad en la asistencia a la mujer en el ciclo gravídico-puerperal; la interconectividad entre saberes y prácticas profesionales.

el significado de la asistencia a la mujer en el ciclo gravídico-puerperal, según la comprensión de los profesionales, está asociado a la aprehensión integral de la mujer – gestante, puérpera, madre, como una unidad indivisible.

## Destacados

**(1)** Se concibe a la gestante y puérpera en la complejidad indivisible de ser mujer y madre.

**(2)** Se sugiere la superación de enfoques reductores y simplificadores.

**(3)** Se señalan rupturas y avances interprofesionales.

**(4)** Se indican movimientos multiprofesionales en la salud materno-infantil.

**(5)** Se sostiene el pensamiento de la complejidad como un referente pertinente y actual.

## Introducción

El ciclo gravídico-puerperal se configura en la vida de una mujer como un recorrido que impone una asistencia singular y multidimensional, por excelencia. Aunque transitorio, este período se desencadena por modificaciones continuas y definitivas en la dinámica y organización del ser mujer/madre, tanto en las dimensiones físicas y emocionales como en las dimensiones socioculturales, que trascienden la maternidad^(2022-2018)^.

Este período implica una dinámica indivisible que requiere, por parte de los profesionales de la salud, una comprensión y asistencia ampliada a las gestantes en las dimensiones física, emocional, social y espiritual. Sin embargo, la asistencia profesional a menudo se reduce únicamente a la dimensión física y sus contornos con la enfermedad, el riesgo de complicaciones y las intervenciones puntuales asociadas con la identificación y prevención de enfermedades^(2021-2022)^.

Estudios señalan que hay poca mención sobre la importancia del manejo de las demandas emocionales, sociales y espirituales de las gestantes y puérperas, debido a los enfoques dicotómicos y fragmentados en el ciclo gravídico-puerperal. Por otro lado, se reconoce que la asistencia integral, continua y complementaria puede minimizar las tensiones emocionales, físicas y mejorar el bienestar de la madre y del hijo^(2023-2020)^. El apoyo social y la atención emocional favorecen el vínculo madre-bebé, y la espiritualidad desempeña un papel esencial en la reducción del estrés, además de posibilitar mecanismos para enfrentarlo^(2021)^.

Por lo tanto, la asistencia ampliada y multiprofesional a la mujer en el ciclo gravídico-puerperal puede dinamizar procesos interactivos, complementarios y sistémicos capaces de involucrar las diferentes áreas y saberes profesionales de salud. Para ello, se requieren referencias que amplíen el pensamiento y que conciban a la mujer, gestante, puérpera, madre como una unidad compleja, no reducida a fases o dimensiones disociables^(2020-2018)^.

Entre los supuestos teóricos del pensamiento de la complejidad se conciben en este estudio la indisociabilidad, la complementariedad y la interconectividad. Estos pilares trascienden el pensamiento cartesiano reduccionista regido por el orden, la separabilidad y la previsibilidad, sacudido por el desarrollo evolutivo de las ciencias. Al aprehender el ciclo gravídico en su unicidad y multidimensionalidad, el pensamiento de la complejidad tiende a superar los enfoques jerárquicos entre saberes, a rescatar las relaciones existentes entre los conocimientos y a posibilitar una asistencia ampliada y multidimensional a la mujer en el ciclo gravídico-puerperal^(2015)^.

En la búsqueda de una comprensión ampliada y multiprofesional, más allá del período gravídico-puerperal del significado de ser mujer/madre, y con el deseo de contribuir al conocimiento propio de la Enfermería en la prospección de un pensamiento que integre, distinga y complemente, se plantea la siguiente pregunta de investigación: ¿cuál es el significado de la asistencia singular y multidimensional a la mujer en el ciclo gravídico-puerperal para los profesionales de la salud? Con base en lo expuesto, el objetivo es comprender el significado de la asistencia a la mujer en el ciclo gravídico-puerperal desde la perspectiva de los profesionales de la salud a la luz del pensamiento de la complejidad.

## Método

### Tipo de estudio

Investigación cualitativa anclada en los supuestos teóricos del pensamiento de la complejidad, tales como: indisociabilidad, complementariedad e interconectividad. El pensamiento complejo posibilita un recorrido metodológico en el que el investigador es inducido a aprender, reinventarse y (re)crearse en el proceso, mediante procesos interpretativos e inductores de avances en la gestión y asistencia en salud. Como método, el pensamiento de la complejidad amplía la comprensión dinámica de ser mujer (gestante, puérpera, madre), permeada por una organización y experiencias singulares entrelazadas, en las cuales la asistencia/cuidado debe considerar la unidad indisociable en las diferentes fases, así como cada fase –gestación, parto, puerperio– en el todo complejo^(2015-2014)^.

### Escenario, participantes y criterios de selección

Participaron en el estudio 23 profesionales que trabajan en Unidades Básicas de Salud, específicamente en el área de salud materno-infantil. Se invitó a todos los profesionales que trabajaban en el área de salud materno-infantil de la región central de Rio Grande do Sul por más de dos años, mediante una invitación formal individual. No obstante, se incluyó solo a profesionales con experiencia comprobada en la asistencia a mujeres en el ciclo gravídico-puerperal. Se excluyeron los profesionales de salud que trabajaban exclusivamente en la gestación o en el puerperio, y los profesionales que estaban ausentes del servicio por algún motivo justificado.

Inicialmente, se estimó un total de 33 participantes con representación regional significativa en el área de salud materno-infantil, pero solo 23 profesionales de la salud cumplieron con los criterios de inclusión y exclusión: 14 enfermeros, seis médicos y tres odontólogos. Los participantes fueron contactados por correo electrónico, a partir de la información proporcionada por la coordinadora regional de salud. Los datos fueron recolectados en el lugar de trabajo de cada participante, en un espacio reservado y señalado por el propio profesional.

### Técnica y período de recolección de datos

Los datos se recolectaron mediante entrevistas individuales, entre marzo y agosto de 2022, realizadas por un enfermero previamente capacitado para este fin. Las entrevistas se basaron en las siguientes preguntas: ¿cuál es el significado de la asistencia ampliada y multiprofesional a la mujer en el ciclo gravídico-puerperal para usted? En su opinión, ¿cómo promover una asistencia complementaria e integradora a la mujer en el ciclo gravídico-puerperal? ¿Cómo avanzar y posibilitar prácticas más interconectadas y horizontalizadas a la mujer en el ciclo gravídico-puerperal? Estas preguntas se profundizaron para abarcar nuevos significados y percepciones.

Las entrevistas individuales fueron realizadas por un solo entrevistador, en el lugar, día y hora indicados por los participantes del estudio. Se priorizó crear un ambiente acogedor y libre de ruidos para asegurar la singularidad y privacidad de cada participante. Las entrevistas fueron grabadas en un grabador digital y tuvieron una duración de aproximadamente 40 minutos. Una vez organizadas, las entrevistas fueron transcritas en su totalidad en un editor de texto por dos investigadores. El conjunto de datos transcritos resultó en 68 páginas, las cuales fueron aprobadas por los entrevistados.

### Tratamiento y análisis de los datos

Los datos se analizaron utilizando la técnica de análisis temático^(2020)^, sistematizada en seis etapas, como sigue: a) ambientación – fase en la que los investigadores se sumergieron profundamente en los datos, familiarizándose ampliamente con los elementos textuales; b) compilación inductiva de códigos iniciales – etapa en la que los investigadores codificaron manual y sistemáticamente el conjunto de datos con atención plena e igual a cada elemento textual; c) generación de temas de significado – etapa en la que los diferentes códigos fueron combinados en temáticas amplias; d) revisión de las temáticas – etapa en la que los investigadores refinaron los temas para señalar, por separado, las similitudes y distinciones entre ellos; e) designación de los temas – fase en la que los investigadores identificaron la esencia/centralidad de cada tema, tanto individualmente como en conjunto; f) elaboración del informe de investigación – fase en la que los investigadores refinaron, en detalle, el conjunto de datos y, finalmente, redactaron el informe. Este proceso de análisis no fue puntual ni lineal, sino que exigió movimientos inductivos y recursivos, como lo propone el pensamiento de la complejidad.

El proceso de análisis de los datos comenzó con las primeras entrevistas, basado en patrones de significado. Para ello, se realizaron registros constantes de ideas, *insights*, borradores y esquemas, no con el objetivo de alcanzar precisión, sino con el fin de lograr profundidad y significado, a la luz del pensamiento de la complejidad.

### Aspectos éticos

Se consideraron, para el cumplimiento de los aspectos éticos, las recomendaciones de la Resolución n.º 466/2012 del Consejo Nacional de Salud. Para mantener el anonimato, las declaraciones de los participantes fueron identificadas a lo largo del texto con la letra “PS” (Profesional de Salud), seguida de un número arábigo de acuerdo con el orden de las declaraciones: PS1... PS23. Los participantes fueron informados sobre los objetivos, la metodología utilizada, su derecho a acceder libremente a los datos y su derecho de retirarse en cualquier momento. Su consentimiento y aceptación para participar en la investigación se obtuvo mediante la firma del Consentimiento Informado. El proyecto fue aprobado por el comité de ética en investigación bajo el número: 5.183.232 y el Certificado de Presentación para Apreciación Ética (CAAE): 54380521.2.0000.5306.

## Resultados

De los 23 participantes, la edad promedio fue de 38 años y todos contaban con educación superior completa. El tiempo de trabajo de los profesionales en el área de salud materno-infantil en el ámbito de la Atención Primaria de Salud oscilaba entre 3 y 19 años.

A continuación, se presentan las tres temáticas a la luz del pensamiento de la complejidad: la indisociabilidad de la asistencia a la gestante y puérpera en la complejidad de ser mujer y madre; la complementariedad en la asistencia a la mujer en el ciclo gravídico-puerperal; la interconectividad entre saberes y prácticas profesionales.

### La indisociabilidad de la asistencia a la gestante y puérpera en la complejidad de ser mujer y madre

Se denotó en las declaraciones de los participantes la necesidad de invertir en la indisociabilidad de la asistencia integral a la mujer, es decir, más allá de las fases singulares de la mujer como la gestación, el parto y el puerperio. Algunos participantes mencionaron que, en ocasiones, el ciclo es fragmentado y se aborda en partes separadas: *Muchas veces, siento que cada profesional percibe y atiende una parte, sin lograr ver que la mujer es la misma, única, en la gestación, el parto, el puerperio y como madre (PS 5).*

El ciclo gravídico-puerperal es considerado por los participantes como un período indivisible y no reducible a consultas, exámenes, prevención de enfermedades o grupos de gestantes, ya que el desenlace favorable o no de una de las fases compromete la dinámica vital de la mujer. Un participante enfatizó la importancia de acoger y comprender a la mujer en su contexto, y que el compromiso de todos los profesionales y sectores debe conducir a la integralidad y complementariedad de la asistencia: *El embarazo, el parto y la llegada del bebé son momentos únicos en la vida de una mujer y su familia. Todo debe funcionar de forma integrada y continua. No se puede asistir a la mujer por etapas, sino acogerla y comprenderla en todas sus dimensiones. La familia debe ser acogida e integrada en todo este proceso* (PS 9).

La calidad de la atención a gestantes y puérperas, según los participantes de este estudio, debe evolucionar y alcanzar un nuevo significado, ya que perciben que la asistencia sigue fragmentada y organizada en fases distintas y puntuales. En este sentido, la calidad se tradujo en la habilidad profesional de crear espacios dialógicos y organizados en redes de atención: *Hemos avanzado mucho en la atención a gestantes y puérperas, pero aún trabajamos aislados y no vemos a la mujer gestante, puérpera, madre como una única entidad integrada en una red* (PS 10). *A veces todo está muy separado y olvidamos que la mujer que recorre los diferentes puntos de la red es la misma* (PS 12).

Algunos participantes expresaron cierta incomodidad y reconocieron claramente las fragilidades en la atención integral a la mujer. Consideran que la calidad de la asistencia implica avanzar en la comprensión teórica y en la capacidad de acoger a la mujer – gestante, puérpera, madre – en su dimensión singular y multidimensional. Al respecto, mencionaron la importancia de espacios concretos de interlocución entre los profesionales de los distintos puntos de la red, con el fin de ampliar la comprensión y discutir estrategias colegiadas de intervención: *Todos deberían tener conciencia de la función de cada profesional en la línea de atención materno-infantil. A veces solo nos preocupamos por nuestra parte y olvidamos que la gestante y la puérpera deben estar en el centro de la atención* (PS 15). *En cuanto a la cesárea, no tenemos un lenguaje unificado. Cada uno lo explica desde su punto de vista y comprensión. Esto debe mejorar mucho para que la mujer realmente ocupe el centro de la atención* (PS 17).

Aunque la mayoría de los participantes destacó avances en la atención a las mujeres durante el prenatal y el parto, un pequeño grupo subrayó la necesidad de mejorar la atención en el puerperio, que generalmente queda relegada a un segundo plano. Un participante destacó el progreso en la humanización del parto, pero señaló que el puerperio no ha avanzado de la misma manera: *Aquí hacemos lo mejor para que la mujer tenga un parto tranquilo y humanizado, pero hablando con una puérpera, ella me dijo que nadie había vuelto a ponerse en contacto con ella después del nacimiento del niño* (PS 23).

En general, los participantes expresaron el deseo de avanzar tanto en la comprensión teórica ampliada como en la atención integral a las mujeres en el ciclo gravídico-puerperal. Si bien algunos no lograron expresarse en términos científicos, reconocen que la mujer-usuaria, gestante, puérpera o no, es un ser singular e integrante de una realidad personal, familiar y social más amplia.

### La complementariedad en la asistencia a la mujer en el ciclo gravídico-puerperal

Varios participantes mencionaron que, por lo general, cada profesional ve y ejecuta solo su parte, su rutina, y, en consecuencia, defiende sus especificidades locales sin considerar la red de salud: *Percibo la falta de alineamiento entre los profesionales que trabajan en la línea de salud materno-infantil. La mujer es la misma y las conductas deben converger para una mejor asistencia* (PS 4).

Un participante, en particular, señaló que la contrarreferencia rara vez ocurre por parte de los profesionales que trabajan en el área hospitalaria. Bajo este enfoque, destacó la falta de comunicación y diálogo entre los diversos sectores y actores de la red. *Ya sucedió que un niño nació con problemas y evolucionó hacia una parada cardiorrespiratoria, y nosotros, en la Unidad de Salud, no fuimos informados. Falta comunicación y comprensión del trabajo de cada profesional. Cada uno cree que está haciendo lo mejor, pero no sabemos lo que pasa del otro lado. Cada uno defiende su parte* (PS 13).

En las declaraciones de los participantes se percibió la relevancia del proceso de trabajo complementario y continuo entre los diversos servicios de la red de salud. Un participante reflexionó que, en el ámbito del Sistema Único de Salud (SUS), nadie trabaja solo o aislado, y que el usuario debe ser acogido como parte de una red, en la que cada punto es complementario al otro. *Debemos entender que en el SUS nada ocurre de forma aislada o separada. La mujer no puede estar deambulando de un servicio a otro. Todos somos responsables, no importa en qué unidad trabaje. Necesitamos ver al SUS como un gran sistema que solo funciona con la cooperación de todos los profesionales de la red* (PS 18).

Un participante, en particular, señaló que más allá del conocimiento técnico, los profesionales necesitan conocer amplia y profundamente el funcionamiento de la red de salud materno-infantil: *Debes conocer cómo funciona la red materno-infantil en su totalidad. Nuestra tendencia, muchas veces, es enfocarnos solo en lo que sucede en nuestro lugar de trabajo sin percibir las consecuencias de cada acto profesional en la vida del usuario. Deberíamos tener más encuentros para estudiar y conocer lo que sucede en los diversos puntos de la red* (PS 19).

En la mayoría de los testimonios se observó la necesidad de desencadenar una nueva forma de pensar y actuar en la asistencia a la mujer en el ciclo gravídico-puerperal. Aunque se reconocen iniciativas y avances en el área, no todos los profesionales están debidamente alineados con los principios y directrices que rigen la atención integral a la salud de la mujer.

### La interconectividad entre saberes y prácticas profesionales

Varios participantes hicieron referencia a la urgencia de promover y ampliar los espacios de interlocución para compartir saberes y prácticas entre los profesionales de los diferentes puntos de la red de salud materno-infantil. Una profesional destacó la escasa interlocución entre los profesionales, sectores y servicios: *Veo que ocurren cosas tan lindas en los servicios, pero no las divulgamos ni nos enteramos. Los logros de un servicio pueden impulsar otras iniciativas, pero a veces no nos enteramos. El otro día, una usuaria me contó todo lo que la unidad hace para que las personas se sientan acogidas* (PS 6).

En otras declaraciones se mencionó que las nuevas evidencias científicas del área no siempre se consideran y aplican en la práctica profesional. En esa línea, algunos participantes señalaron que hay profesionales que orientan su práctica en modelos tradicionales, es decir, sin criterios ni evidencias científicas. *Hay profesionales que siguen defendiendo prácticas tradicionales que hoy no tienen ningún sentido. Muchas de ellas incluso están contraindicadas. Veo esto como un gran desafío. Las cosas cambian, y nuestra mentalidad también debe evolucionar* (PS 14).

Otros participantes también señalaron la importancia de la formación profesional prospectiva. Reconocen que no siempre los profesionales de la salud se forman bajo una lógica de cooperación e interconectividad entre saberes y prácticas basadas en las mejores evidencias científicas. En este sentido, indicaron que superar los procesos fragmentados y dicotómicos necesariamente implica una formación profesional que amplíe las perspectivas multiprofesionales. *La formación profesional debe evolucionar en muchos aspectos. La teoría no siempre tiene sentido en la práctica, así como la práctica sin teoría no tiene significado. ¿Cómo deben evolucionar las cosas? Yo creo que debe haber más diálogo e interlocución entre las universidades y los servicios* (PS 20).

La interlocución necesaria también se demostró en la actuación multiprofesional e interprofesional. Para la mayoría de los participantes, la calidad de la asistencia a la mujer en el ciclo gravídico-puerperal requiere una percepción ampliada y una actuación multiprofesional, a partir de la inducción de espacios compartidos para la construcción del conocimiento y la toma de decisiones colegiadas. *Debemos aprender unos de otros. Una asistencia de calidad no depende del trabajo de un solo profesional o servicio. Cada profesional tiene su percepción, y juntos podemos lograr más* (PS 21).

La educación continua en salud se constituye, según los participantes, en un importante dispositivo que agrega y dinamiza posturas profesionales más dialógicas, colaborativas y complementarias entre los profesionales que integran la red de salud materno-infantil. Además de la habilidad y competencia técnica, es fundamental que los profesionales de salud sean capaces de trascender su propio saber y su espacio de práctica profesional.

## Discusión

El pensamiento de la complejidad induce a tejer, conjuntamente, el entramado de hilos que integran, complementan y dinamizan la asistencia a la gestante y puérpera en la complejidad indivisible de ser mujer y madre. Las diversas fases y experiencias de la mujer se articulan para producir un todo que repercute sobre las diferentes dimensiones^(2015-2014)^. Así, el pensamiento de la complejidad representa un desafío para el conocimiento y un impulso hacia la evolución prospectiva en el ámbito de la salud materno-infantil.

En lugar de concebir y promover una asistencia fragmentada en fases – gestación, parto, puerperio, maternidad –, la mujer debe ser comprendida en su realidad existencial indivisible, la cual requiere un conocimiento ampliado, singular, complementario e interconectado con los hechos, experiencias y trayectorias que amplían perspectivas y fortalecen vínculos^(2023-2020)^. La asistencia ampliada e integral a la mujer en el ciclo gravídico-puerperal necesita, bajo esta mirada multifactorial y prospectiva, contemplar enfoques y prácticas complementarias y multiprofesionales, con el objetivo de alcanzar las dimensiones física, psíquica, social y espiritual.

Las evidencias científicas corroboran esta visión ampliada y evolutiva al demostrar la necesidad de centrar la atención en la mujer como una unidad compleja, independientemente de la fase o período en que se encuentre. Al abarcar la complejidad del cuidado/asistencia, los profesionales reconocen el derecho de la mujer a tomar decisiones autónomas y facilitan trayectorias complementarias e indisociables en el ciclo gravídico-puerperal^(2024-2022)^.

Avanzar hacia los supuestos teóricos del pensamiento de la complejidad es esencial para que, frente a los desafíos del modelo hegemónico de intervención en el área de la salud materno-infantil, se promueva una visión compleja del mundo, del ser humano y del cuidado en salud desde una perspectiva interprofesional. Superar los enfoques prevalentes en la asistencia a la mujer en el ciclo gravídico-puerperal requiere, por lo tanto, una percepción ampliada y multiprofesional, basada en espacios compartidos para la construcción del conocimiento^(2024)^.

Al reconocer que “*la mujer no puede ser asistida en etapas*”, se concibe que el todo va más allá de la simple suma de las partes/períodos del ciclo gravídico-puerperal. A medida que las partes se integran y complementan para formar el todo, se generan nuevas ideas, comportamientos y evoluciones teórico-prácticas. Se abre espacio al intercambio de conocimientos, a la interconectividad de los diálogos y a la indisociabilidad de los enfoques y prácticas profesionales^(2023)^.

Los avances técnico-científicos dependen, cada vez más, del conocimiento articulado y compartido entre profesionales de diversas áreas. Es necesario fomentar referentes que trasciendan las posturas profesionales tradicionales e inflexibles aún presentes en los escenarios de salud, como se evidenció en este estudio: “*ellos siguen defendiendo sus prácticas tradicionales, que hoy no tienen ningún sentido*”. Más allá de las nuevas políticas y acciones estratégicas dirigidas a gestantes y puérperas, son cada vez más necesarias nuevas conductas y actitudes individuales y colectivas, basadas en enfoques compartidos y respaldadas por las mejores evidencias científicas^(2023-2023)^.

¿Cómo, entonces, abrir nuevas posibilidades teóricas y promover inversiones profesionales que contemplen la asistencia a la mujer en su unicidad y multidimensionalidad? Fomentar conocimientos y prácticas más avanzadas y complejas en el área de la salud implica necesariamente avanzar en el pensamiento sistémico y reconocer enfoques interprofesionales y multidimensionales capaces de trascender la fragmentación y la linealidad del ser y promover la asistencia a la mujer en el ciclo gravídico-puerperal^(2022-2020)^.

Concebir la calidad de la asistencia en el ciclo gravídico-puerperal dentro de la complejidad indivisible de ser mujer y madre remonta, en la lógica del pensamiento de la complejidad, a una tradición histórico-hegemónica en la cual prevalecieron relaciones verticalizadas, prescriptivas y previsibles, que a menudo desconsideraban la autonomía y la toma de decisiones de la mujer^(2022)^. Bajo este enfoque hegemónico tradicional, la mujer solía ser privada de sus derechos y rara vez era alentada a ser protagonista y tomar decisiones autónomas^(2023)^. Este pensamiento y acción tradicional-reductora no se alinea con los supuestos teóricos de la indisociabilidad, complementariedad e interconectividad a la luz del pensamiento de la complejidad.

Fenómenos complejos emergen del comportamiento dinámico de las unidades interactivas y asociativas referidas como autoorganizadas, que ocurren en sistemas que son disipativos y no lineales^(2023)^. Bajo este enfoque, el conocimiento del todo depende de las partes, al igual que el conocimiento de las partes depende del todo, como lo demostraron los participantes. Este proceso refleja la capacidad profesional de interconectar diferentes áreas y servicios que componen la red de salud materno-infantil y, consecuentemente, dialogar con los diversos saberes profesionales.

El ser humano – mujer – es, por excelencia, una unidad compleja, es decir, productora y producto de una complejidad dinámica, evolutiva y multidimensional^(2021)^. Así, aunque se niegue la indisociabilidad, la complementariedad y la interconectividad en el ciclo gravídico-puerperal, la asistencia integral a la mujer – gestante, puérpera, madre – implica necesariamente la ruptura de modelos hegemónicos y la reforma del pensamiento por parte de los profesionales de la salud. No es suficiente tener una red organizada y articulada en salud materno-infantil si, paralelamente, el pensamiento y la actuación de los profesionales de la salud siguen siendo verticales, fragmentados y basados en saberes disciplinares y unidimensionales.

Evolucionar en el pensamiento significa, desde una perspectiva evolutiva y prospectiva, comprender a la mujer en su unicidad y complementariedad; trascender las barreras disciplinarias del área de la salud y alcanzar un pensamiento integrador, colaborativo e indisociable^(2015-2014)^. Este camino evolutivo implica reconocer que la calidad de la atención a la mujer está asociada a la capacidad de evolucionar en la reflexividad, que involucra acciones contradictorias y aparentemente antagónicas para generar fenómenos complejos y sistémicos.

Las contribuciones de este estudio para el avance del conocimiento científico en el área de la salud materno-infantil están relacionadas con la superación de un pensamiento reduccionista que concibe a la mujer (gestante, puérpera, madre) en partes fragmentadas y disociables. La asistencia integral y de calidad a la mujer en su ciclo gravídico-puerperal implica necesariamente rupturas y avances, los cuales pueden ser liderados por enfermeros imbuidos de un pensamiento amplio, integrador y prospectivo. Es fundamental que el enfermero se empodere de su conocimiento singular y contribuya, de manera prospectiva, a una actuación evolutiva en el contexto de la salud materno-infantil.

Se considera, como limitación de este estudio, la no inclusión de la totalidad de los participantes representativos en el área, previamente previstos e incluidos en el estudio. Otra limitación está asociada al poco tiempo disponible para que los participantes realizaran las entrevistas con el significado y la profundidad necesarios.

## Conclusión

El significado de la asistencia a la mujer en el ciclo gravídico-puerperal, según la comprensión de los profesionales, está asociado a la aprehensión integral de la mujer – gestante, puérpera, madre – como una unidad indivisible. Más allá del ciclo gravídico-puerperal, la mujer debe ser comprendida en sus vivencias y experiencias singulares e indivisibles.

La aprehensión ampliada y contextualizada de la asistencia/cuidado, como una unidad compleja – singular y multidimensional – es imprescindible para fomentar la autonomía, la creatividad, la interactividad, y en definitiva, las relaciones cercanas, dialógicas y humanizadas en el contexto de la salud materno-infantil.

Esta investigación resalta la importancia y recomienda nuevos estudios sobre la necesaria evolución del pensamiento en la asistencia a las gestantes y puérperas, con el fin de promover enfoques integradores y colaborativos entre los profesionales y con los usuarios de los servicios de salud.

# Translation: Assistência à mulher no ciclo gravídico-puerperal na perspectiva de profissionais de saúde à luz do pensamento da complexidade*

**DOI:** 10.1590/1518-8345.7313.4459

**Authors:** Dirce Stein Backes, Taina Ribas de Morais, Caroline Brondani Rosa, Leris Salete Bonfanti Haeffner, Dulce Maria Pereira Garcia Galvão, Adriana Dall’Asta Pereira

**Affiliations:** 1 Universidade Franciscana, Santa Maria, RS, Brasil; 2 Bolsista do Conselho Nacional de Desenvolvimento Científico e Tecnológico (CNPq), Brasil; 3 Bolsista da Coordenação de Aperfeiçoamento de Pessoal de Nível Superior (CAPES), Brasil; 4 Escola Superior de Enfermagem, Coimbra, Portugal

**Keywords:** Enfermagem, Atendimento Integral à Saúde da Mulher, Gestação, Puerpério, Atenção Primária à Saúde, Pesquisa Qualitativa

## Abstract

compreender o significado da assistência à mulher no ciclo gravídico-puerperal, na perspectiva de profissionais de saúde à luz do pensamento da complexidade.

pesquisa qualitativa ancorada em pressupostos teóricos do pensamento da complexidade: indissociabilidade, complementariedade e interconectividade. Participaram 23 profissionais da área de saúde materno-infantil, da região sul do Brasil. Os dados foram coletados por meio de entrevista individual e analisados pela técnica de análise temática.

as três temáticas de análise foram a indissociabilidade da assistência à gestante e puérpera na complexidade de ser mulher e mãe; a complementariedade na assistência à mulher no ciclo gravídico-puerperal; a interconectividade entre saberes e práticas profissionais.

o significado da assistência à mulher no ciclo gravídico-puerperal, na compreensão dos profissionais, está associado à apreensão integral da mulher – gestante, puérpera, mãe, como unidade indivisível.

## Destaques

**(1)** Concebe-se a gestante e puérpera na complexidade indivisível de ser mulher e mãe.

**(2)** Sugere-se a superação de abordagens redutoras e simplificadoras.

**(3)** Sinaliza-se rupturas e avanços interprofissionais.

**(4)** Indica-se movimentos multiprofissionais na saúde materno-infantil.

**(5)** Sustenta-se o pensamento da complexidade como referencial pertinente e atual.

## Introdução

O ciclo gravídico-puerperal configura-se na vida de uma mulher como um percurso que impõe assistência singular e multidimensional, por excelência. Embora transitório, esse período é desencadeado por modificações continuas e definitivas na dinâmica e na organização do ser mulher/mãe tanto nas dimensões física e emocional quanto nas dimensões socioculturais, as quais transcendem a maternidade^(2022-2018)^.

O referido período envolve uma dinâmica indivisível que requer, por parte dos profissionais de saúde, compreensão e assistência ampliada às gestantes nas dimensões física, emocional, social e espiritual. No entanto, a assistência profissional reduz-se, frequentemente, apenas à dimensão física e seus contornos com a doença, risco de complicações e intervenções pontuais associadas à identificação e à prevenção de doenças^(2021-2022)^.

Estudos denotam que há pouca menção à importância do manejo das demandas emocionais, sociais e espirituais das gestantes e puérperas, em decorrência das abordagens dicotômicas e fragmentadas no ciclo gravídico-puerperal. Por outro lado, reconhecem que a assistência integral, continuada e complementar é capaz de minimizar tensões emocionais, físicas e melhorar o bem-estar da mãe e do filho^(2023-2020)^. O suporte social e a atenção emocional favorecem o vínculo mãe-bebê e a espiritualidade desempenha papel essencial na redução do estresse, além de possibilitar mecanismos para o seu enfrentamento^(2021)^.

Portanto, a assistência ampliada e multiprofissional à mulher no ciclo gravídico-puerperal pode dinamizar processos interativos, complementares e sistêmicos capazes de envolver as diferentes áreas e saberes profissionais de saúde. Requer-se, para tanto, referenciais que ampliem o pensamento e que concebam a mulher, gestante, puérpera, mãe como unidade complexa, não reduzida a fases ou dimensões dissociáveis^(2020-2018)^.

Dentre os pressupostos teóricos do pensamento da complexidade concebe-se, neste estudo, a indissociabilidade, a complementariedade e a interconectividade. Esses pilares transcendem o pensamento cartesiano reducionista regido pela ordem, a separabilidade e a previsibilidade, abalado pelo desenvolvimento evolutivo das ciências. Ao apreender o ciclo gravídico em sua unicidade e multidimensionalidade, o pensamento da complexidade tende a superar abordagens hierárquicas entre saberes, resgatar as relações existentes entre os conhecimentos e possibilitar uma assistência ampliada e multidimensional à mulher no ciclo gravídico-puerperal^(2015)^.

Na busca pela compreensão ampliada e multiprofissional, para além do período gravídico-puerperal do significado de ser mulher/mãe, e no desejo de contribuir com o saber próprio da Enfermagem na prospecção de um pensamento que integra, distingue e complementa, tem-se como questão pesquisa: qual o significado da assistência singular e multidimensional à mulher no ciclo gravídico-puerperal para profissionais da saúde? Objetiva-se, com base no exposto, compreender o significado da assistência à mulher no ciclo gravídico-puerperal, na perspectiva de profissionais de saúde à luz do pensamento da complexidade.

## Método

### Tipo de estudo

Pesquisa qualitativa ancorada em pressupostos teóricos do pensamento da complexidade, tais como: indissociabilidade, complementariedade e interconectividade. O pensamento complexo possibilita um percurso metodológico em que o pesquisador é induzido a aprender, a inventar-se e a (re)criar-se no percurso, mediante processos interpretativos e indutores de avanços em âmbito da gestão e da assistência à saúde. Como método, o pensamento da complexidade amplia a compreensão dinâmica de ser mulher (gestante, puérpera, mãe), permeada por uma organização e vivências singulares tecidas em conjunto, na qual a assistência/cuidado necessita considerar a unidade indissociável nas diferentes fases, assim como cada fase – gestação, parto, puerpério – no todo complexo^(2015-2014)^.

### Cenário, participantes e critérios de seleção

Participaram do estudo 23 profissionais que atuam em Unidades Básicas de Saúde, mais especificamente, na área saúde materno-infantil. Convidou-se, por meio de convite formal individual, todos os profissionais que atuavam na área saúde materno-infantil da região central do Rio Grande do Sul, por mais de dois anos. Incluiu-se, no entanto, profissionais com experiência comprovada na assistência à mulher no ciclo gravídico-puerperal. Foram critérios de exclusão: profissionais de saúde que atuavam apenas na gestação ou no puerpério, e profissionais afastados do serviço por algum motivo justificado.

Estimou-se, inicialmente, um total de 33 participantes com representação regional significativa na área saúde materno-infantil, mas atenderam aos critérios de inclusão e exclusão apenas 23 profissionais de saúde, quais sejam: 14 enfermeiros, seis médicos e três odontólogos. Os participantes foram acessados por *e-mail*, a partir do contato fornecido pela coordenadora regional de saúde. Os dados foram coletados no local de atuação de cada participante, em espaço reservado e indicado pelo profissional.

### Técnica e período de coleta dos dados

Os dados foram coletados por meio de entrevistas individuais, entre março e agosto de 2022, por um profissional Enfermeiro previamente instrumentalizado para este fim. As entrevistas foram conduzidas com base nas seguintes questões: qual o significado da assistência ampliada e multiprofissional à mulher no ciclo gravídico-puerperal para você? Em sua opinião, como promover uma assistência complementar e integradora à mulher no ciclo gravídico-puerperal? Como avançar e possibilitar práticas mais interconectadas e horizontalizadas à mulher no ciclo gravídico-puerperal? Essas questões foram aprofundadas de modo a contemplar novos significados e percepções.

As entrevistas individuais foram realizadas por apenas um entrevistador, em local, dia e horário indicado pelos participantes do estudo. Primou-se, nesse percurso, pela ambiência acolhedora e livre de ruídos, de modo a assegurar a singularidade e a privacidade de cada participante. As entrevistas foram gravadas em gravador digital e tiveram uma duração de aproximadamente 40 minutos. Após organizadas, as entrevistas foram transcritas integralmente em editor de texto por dois pesquisadores. O conjunto de dados transcritos resultou em 68 páginas, as quais passaram pela aprovação dos entrevistados.

### Tratamento e análise dos dados

Os dados foram analisados com base na técnica de análise temática^(2020)^, sistematizada em seis etapas, conforme segue: a) ambientação – fase em que os pesquisadores imergiram profundamente nos dados, no sentido de familiarizar-se amplamente com os elementos textuais; b) compilação indutiva de códigos iniciais – etapa em que os pesquisadores codificaram, de forma manual e sistemática, o conjunto de dados com atenção plena e igual a cada elemento textual; c) geração de temas de significação – etapa em que os diferentes códigos foram combinados em temáticas abrangentes; d) revisão das temáticas – etapa em que os pesquisadores refinaram os temas, a fim de denotar, separadamente, as proximidades e as distinções entre os temas; e) designação dos temas – fase em que os pesquisadores identificaram a essência/centralidade de cada temática, em particular e no conjunto dos temas; f) elaboração do relatório de pesquisa – fase em que os pesquisadores refinaram, em detalhes, o conjunto de dados e, por fim, escreveram o relatório. Esse processo de análise não foi, no entanto, pontual e linear, mas demandou movimentos indutivos e recursivos, conforme proposto pelo pensamento da complexidade.

O processo de análise dos dados ocorreu desde a condução das primeiras entrevistas, com base em padrões de significados. Realizou-se, para tanto, o registro constante de ideias, insights, rascunhos e esquemas, não com o objetivo de alcançar acurácia, mas no intuito de alcançar profundidade e significado, à luz do pensamento da complexidade.

### Aspectos éticos

Considerou-se, para o cumprimento das questões éticas, as recomendações da Resolução n° 466/2012 do Conselho Nacional de Saúde. Para manter o anonimato, as falas dos participantes foram identificadas, ao longo do texto, pela letra “PS” (Profissional de Saúde), seguida de um algarismo arábico de acordo com a ordem das falas: PS1... PS23. Os participantes da pesquisa foram esclarecidos sobre as finalidades, a metodologia utilizada, o direito de livre acesso aos dados e a sua desistência a qualquer momento. O seu consentimento e aceite à pesquisa se deu por meio da assinatura do Termo de Consentimento Livre e Esclarecido. O projeto foi aprovado pelo comitê de ética em pesquisa sob o número: 5.183.232 e Certificado de Apresentação de Apreciação Ética (CAAE): 54380521.2.0000.5306.

## Resultados

Dos 23 participantes, a média de idade foi de 38 anos e, de escolaridade, o ensino superior completo. O período de atuação dos profissionais na área saúde materno-infantil em âmbito da Atenção Primária à Saúde foi de 3 a 19 anos.

Apresenta-se, a seguir, as três temáticas à luz do pensamento da complexidade: a indissociabilidade da assistência à gestante e puérpera na complexidade de ser mulher e mãe; a complementariedade na assistência à mulher no ciclo gravídico-puerperal; a interconectividade entre saberes e práticas profissionais.

### A indissociabilidade da assistência à gestante e puérpera na complexidade de ser mulher e mãe

Denotou-se, na fala dos participantes, a necessidade de investir na indissociabilidade da assistência integral à mulher, isto é, para além das fases singulares da mulher como a gestação, o parto e o puerpério. Alguns participantes mencionaram que, por vezes, o ciclo é fragmentado e conduzido em partes separáveis: *Muitas vezes, eu sinto, que cada profissional percebe e cuida de uma parte e não consegue perceber que a mulher é a mesma, é única na gestação, parto, puerpério e mãe* (PS 5).

O ciclo gravídico-puerperal é considerado, pelos participantes, em período indivisível e não reduzível às consultas, exames, prevenção de doenças ou grupos de gestantes, visto que o desfecho favorável e/ou não de uma das partes compromete a dinâmica da mulher em sua organização vital. Um participante enfatizou, de forma mais incisiva, que a mulher precisa ser acolhida e compreendida em seu contexto situacional e que o engajamento de todos os profissionais e setores deve conduzir à integralidade e à complementariedade da assistência: *A gravidez, o parto, a vinda do bebê são momentos únicos na vida de uma mulher e família. Tudo precisa funcionar de forma integrada e continuada. A mulher não pode ser assistida em etapas, mas acolhida e compreendida em todas as dimensões. A família deve ser acolhida e integrada em todo esse processo* (PS 9).

A qualidade da atenção às gestantes e puérperas necessita, na fala dos participantes deste estudo, evoluir e alcançar um novo significado, ao considerarem que a assistência segue fragmentada e organizada em fases distintas e pontuais. Nesse sentido, a qualidade foi traduzida na habilidade profissional de possibilitar espaços dialógicos e organizados em redes de cuidado: *Nota-se que nós evoluímos muito na atenção à gestante e puérpera, mas ainda trabalhamos isolados e não percebemos a mulher gestante, puérpera, mãe como única e integrante de uma rede (PS 10). Às vezes tudo fica muito separado e a gente esquece que a mulher que percorre os diferentes pontos da rede é a mesma* (PS 12).

Alguns participantes referiram certo desconforto e reconhecem, claramente, as fragilidades presentes na assistência integral à mulher. Percebem que a qualidade da assistência consiste em evoluir na compreensão teórica e no exercício de acolher a mulher – gestante, puérpera, mãe em sua dimensão singular e multidimensional. A esse propósito, fizeram referência aos espaços concretos de interlocução entre os profissionais dos diversos pontos da rede, a fim de ampliarem a compressão e discutirem estratégias colegiadas de intervenção: *Todos deveriam ter consciência da função de cada profissional na linha saúde materno-infantil. Às vezes nos preocupamos apenas com a nossa parte e esquecemos que a gestante e a puérpera devem estar no centro da assistência* (PS 15). *Em relação à cesariana não temos uma linguagem única. Cada um explica do seu jeito e a partir da sua visão e compreensão. Isto precisa melhorar muito para que efetivamente a mulher ocupe a centralidade na assistência* (PS 17).

Embora a maioria dos participantes tenha sinalizado avanços na atenção à mulher no período pré-natal e parto, um pequeno grupo reforçou a necessidade de qualificar a assistência puerperal, geralmente, relegada a um segundo plano. No depoimento de um participante ficou notório o avanço conquistado em relação à humanização do parto, mas em contrapartida o puerpério não avançou na mesma proporção: *Aqui fazemos o melhor para que esta mulher tenha um parto tranquilo e humanizado, mas conversando com uma puérpera ela me disse que ninguém mais havia feito contato com ela depois do nascimento da criança* (PS 23).

Denotou-se, na fala dos participantes, em geral, que existe um desejo de evolução tanto no que se refere à compreensão teórica ampliada quanto na assistência integral às mulheres no ciclo gravídico-puerperal. Os participantes não conseguiram, por vezes, expressar-se cientificamente, mas reconhecem que a mulher-usuária, gestante, puérpera ou não, é um ser singular e integrante de uma realidade pessoal, familiar e social ampliada.

### A complementariedade na assistência à mulher no ciclo gravídico-puerperal

Diversos participantes referiram que, geralmente, cada profissional vê e executa apenas a sua parte, a sua rotina e, consequentemente, defende as suas especificidades locais sem considerar a rede de saúde: *Percebo a falta de alinhamentos dos profissionais que atuam na linha de saúde materno-infantil. A mulher é a mesma e as condutas precisam convergir para uma melhor assistência* (PS 4).

Um participante, em especial, denotou que a contrarreferência raramente ocorre por parte dos profissionais que atuam na área hospitalar. Sob esse enfoque, destacou a falta de comunicação e diálogo entre os diversos setores e atores da rede. *Já aconteceu de uma criança nascer com problemas e ter evoluído para uma parada cardiorrespiratória e nós da Unidade de Saúde não tomarmos ciência. Falta comunicação e compressão do trabalho de cada profissional. Cada um acha que está fazendo o melhor, mas não sabemos o que acontece do outro lado. Cada um defende a sua parte* (PS 13).

Percebeu-se, na fala dos participantes, a relevância do processo de trabalho complementar e continuado entre os diversos serviços da rede de saúde. Sob esse enfoque, um participante refletiu que, no âmbito do Sistema Único de Saúde (SUS), ninguém trabalha sozinho ou isolado e que o usuário deve ser acolhido como singular e integrante de uma rede, em que cada ponto é complementar ao outro. *Nós precisamos entender que no SUS nada acontece de forma isolada ou separada. A mulher não pode ficar perambulando de um serviço para o outro. Todos somos responsáveis, não importa em que unidade eu trabalho. Precisamos olhar para o SUS como um grande sistema, que só funciona em cooperação com todos os profissionais da rede* (PS 18).

Um participante, em especial, demonstrou que para além do conhecimento técnico, os profissionais necessitam conhecer ampla e profundamente o funcionamento da rede de saúde materno-infantil: *Você precisa conhecer o funcionamento da rede materno-infantil como um todo. A nossa tendência, muitas vezes, é olhar só o que acontece no próprio local de trabalho sem perceber as consequências de cada ato profissional na vida do usuário. Nós teríamos que ter mais encontros para estudar e conhecer o que acontece nos diversos pontos da rede* (PS 19).

Percebeu-se, na maioria dos depoimentos, a necessidade de desencadear um novo pensar e agir na assistência à mulher no ciclo gravídico-puerperal. Embora reconheçam iniciativas e avanços na área, nem todos os profissionais estão devidamente apropriados e sintonizados com os princípios e diretrizes que regem a atenção integral à saúde da mulher.

### A interconectividade entre saberes e práticas profissionais

Diversos participantes fizeram referência à premência de promover e ampliar os espaços de interlocução para o compartilhamento de saberes e práticas entre os profissionais dos diferentes pontos da rede de saúde materno-infantil. Na fala de uma profissional, mais especificamente, ficou notória a pouca interlocução entre os profissionais, setores e serviços, ao mencionar: *Eu vejo que ocorrem coisas tão lindas nos serviços, mas a gente não divulga e não fica sabendo. As conquistas de um serviço podem impulsionar outras iniciativas, mas as vezes a gente não fica sabendo. Outro dia uma usuária me contou tudo o que a unidade promove para que as pessoas se sentissem acolhidas* (PS 6).

Denotou-se, em outras falas, que as novas evidências científicas da área nem sempre são consideradas e aplicadas na prática profissional. Nessa direção, alguns participantes fizeram referência aos profissionais que orientam a sua prática em modelos tradicionais, isto é, sem critérios e evidências científicas. *Tem profissionais que seguem defendendo as suas práticas tradicionais, que hoje não fazem o mínimo sentido. Muitas delas são até contraindicadas. Eu vejo isto como grande desafio. As coisas mudam e nossa cabeça também precisa evoluir* (PS 14).

Outros participantes, ainda, fizeram referência à formação profissional prospectiva. Reconhecem que nem sempre os profissionais de saúde são formados na lógica da cooperação e interconectividade entre saberes e práticas sustentadas nas melhores evidências científicas. Denotaram, sob esse enfoque, que a superação de processos fragmentados e dicotômicos perpassa necessariamente pela formação profissional pautada em referenciais que ampliam perspectivas multiprofissionais. *A formação profissional precisa evoluir em muitos pontos. A teoria nem sempre tem significado na prática, assim como a prática sem a teoria não tem sentido. Como as coisas devem evoluir? Eu entendo que precisa ter mais diálogo e interlocução entre as universidades e os serviços* (PS 20).

A necessária interlocução foi demonstrada, igualmente, na atuação multiprofissional e interprofissional. Para a maioria dos participantes, a qualidade da assistência à mulher no ciclo gravídico-puerperal requer percepção ampliada e atuação multiprofissional, a partir da indução de espaços compartilhados de construção do conhecimento e à tomada de decisões colegiadas. *Nós precisamos aprender uns com os outros. Uma assistência de qualidade não se dá pelo trabalho de uma única área profissional ou serviço. Cada profissional tem a sua percepção e juntos teremos um maior alcance* (PS 21).

A educação permanente em saúde constitui-se, na visão dos participantes, em um importante dispositivo agregador e dinamizador de posturas profissionais mais dialógicas, colaborativas e complementares entre os profissionais que integram a rede de saúde materno-infantil. Além de habilidade e competência técnica, é fundamental que os profissionais de saúde sejam capazes de transcender o seu saber e o seu espaço de prática profissional.

## Discussão

O pensamento da complexidade induz à tessitura, em conjunto, do emaranhado de fios que integralizam, complementam e dinamizam a assistência à gestante e puérpera na complexidade indivisível de ser mulher e mãe. As diversas fases e vivências da mulher articulam-se para produzir o todo que retroage sobre as diferentes dimensões^(2015-2014)^. Logo, o pensamento da complexidade é um desafio ao conhecimento e um impulso à evolução prospectiva na área da saúde materno-infantil.

Ao invés de conceber e promover uma assistência em fases – gestação, parto, puerpério, mãe, a mulher precisa ser apreendida em sua realidade existencial indivisível, a qual comporta um conhecimento ampliado, singular, complementar e interconectado aos fatos, vivências e percursos que ampliam perspectivas e fortalecem vínculos^(2023-2020)^. A assistência ampliada e integral à mulher no ciclo gravídico-puerperal necessita, sob esse olhar multifatorial e prospectivo, contemplar abordagens e práticas complementares e multiprofissionais, com vistas ao alcance da dimensão física, psíquica, social e espiritual.

Evidências científicas corroboram esse pensar ampliado e evolutivo ao demonstrarem a necessidade de centrar a assistência na mulher – unidade complexa, independente da fase ou período em que se encontra. Ao abarcar a complexidade do cuidado/assistência, os profissionais reconhecem o direito da mulher em relação às decisões autônomas e possibilitam percursos complementares e indissociáveis no ciclo gravídico-puerperal^(2024-2022)^.

Avançar em direção aos pressupostos teóricos do pensamento da complexidade é premente para que, face aos desafios relacionados ao modelo hegemônico de intervenção na área da saúde materno-infantil, se favoreça uma visão complexa de mundo, de ser humano, de cuidado em saúde na perspectiva interprofissional. A superação de abordagens prevalentes na assistência à mulher no ciclo gravídico-puerperal requer, portanto, percepção ampliada e multiprofissional, a partir de espaços compartilhados de construção do conhecimento^(2024)^.

Ao reconhecer que a “*mulher não pode ser assistida em etapas*” concebe-se que o todo vai muito além da simples soma das partes/períodos do ciclo gravídico-puerperal. Assim, na medida em que as partes se integram e complementam para formar o todo, produz-se novas ideias, comportamentos e evoluções teórico-práticas. Dá-se espaço ao compartilhamento de saberes, à interconectividade de diálogos e à indissociabilidade de abordagens e práticas profissionais^(2023)^.

Os avanços técnico-científicos dependem, cada vez mais, do conhecimento articulado e compartilhado entre profissionais das diversas áreas do conhecimento. Requer-se fomentar, nesse sentido, referenciais que transcendam posturas profissionais tradicionais e inflexíveis ainda presentes nos cenários de saúde, conforme evidenciado neste estudo “*elesseguem defendendo as suas práticas tradicionais, que hoje não fazem o mínimo sentido*”. Para além de novas políticas e ações estratégicas voltadas às gestantes e puérperas, são necessárias, crescentemente, novas condutas e atitudes individuais e coletivas, embasadas em abordagens compartilhadas e apoiadas nas melhores evidências científicas^(2023-2023)^.

Como, no entanto, descortinar novas possibilidades teóricas e promover investimentos profissionais que contemplem a assistência à mulher em sua unicidade e multidimensionalidade? O fomento de saberes e práticas mais evoluídos e complexos na área da saúde implica, necessariamente, em evoluir no pensamento sistêmico e reconhecer abordagens interprofissionais e multidimensionais capazes de transcender a fragmentação e a linearidade do ser e promover a assistência à mulher no ciclo gravídico-puerperal^(2022-2020)^.

Conceber a qualidade da assistência no ciclo gravídico-puerperal na complexidade indivisível de ser mulher e mãe remonta, na lógica do pensamento da complexidade, uma tradição histórico-hegemônica, na qual prevaleceram relações verticalizadas, prescritivas e previsíveis, nas quais se desconsidera, frequentemente, a autonomia e a decisão da mulher^(2022)^. Sob esse viés hegemônico tradicional, a mulher era geralmente privada de seus direitos e raramente estimulada ao protagonismo e à tomada de decisões autônomas^(2023)^. Esse pensar e agir tradicional-redutor não pactua com os pressupostos teóricos da indissociabilidade, complementariedade e interconectividade à luz do pensamento da complexidade.

Fenômenos complexos emergem do comportamento dinâmico das unidades interativas e associativas referidas como auto-organizadas, que ocorrem em sistemas que são dissipativos e não lineares^(2023)^. Sob esse pensar, o conhecimento do todo depende das partes, assim como o conhecimento das partes depende do todo, conforme demonstrado pelos participantes. Esse processo reflete a capacidade profissional de interligar diferentes áreas e serviços que compõem a rede de saúde materno-infantil e, consequentemente, dialogar com os diversos saberes profissionais.

O ser humano – mulher – é, por excelência, uma unidade complexa, isto é, produtora e produto de uma complexidade dinâmica, evolutiva e multidimensional^(2021)^. Assim, embora se negue a indissociabilidade, a complementaridade e a interconectividade no ciclo gravídico-puerperal, a assistência integral à mulher – gestante, puérpera, mãe – implica necessariamente na ruptura de modelos hegemônicos e na reforma do pensamento por parte dos profissionais de saúde. Não basta que se tenha uma rede organizada e articulada em saúde materno-infantil se, paralelamente, o pensamento e a atuação dos profissionais de saúde seguirem verticalizados, fragmentados e pautados em saberes disciplinares e unidimensionais.

Evoluir no pensamento significa, na perspectiva evolutiva e prospectiva, apreender a mulher em sua unicidade e complementariedade; transcender as barreiras disciplinares da área da saúde e alcançar um pensamento integrador, colaborativo e indissociável^(2015-2014)^. Esse percurso evolutivo implica em reconhecer que a qualidade da assistência à mulher está associada à capacidade de evoluir na reflexibilidade que comporta ações contraditórias e aparentemente antagônicas para gerar fenômenos complexos e sistêmicos.

As contribuições deste estudo para o avanço do conhecimento científico na área da saúde materno-infantil estão associadas à superação de um pensamento redutor que concebe a mulher (gestante, puérpera, mãe) em partes fragmentadas e dissociáveis. A assistência integral e de qualidade à mulher em seu ciclo gravídico-puerperal implica necessariamente em rupturas e avanços, as quais podem ser protagonizadas pelos Enfermeiros imbuídos de um pensamento ampliado, integrador e prospectivo. É fundamental que o enfermeiro se empodere do seu saber singular e contribua, prospectivamente, para uma atuação evolutiva no contexto da saúde materno-infantil.

Considera-se, como limitação deste estudo, a não totalidade dos participantes representativos na área e previamente previstos e incluídos no estudo. Outra limitação está associada ao pouco tempo dos participantes para a realização das entrevistas com significado e profundidade.

## Conclusão

O significado da assistência à mulher no ciclo gravídico-puerperal, na compreensão dos profissionais, está associado à apreensão integral da mulher – gestante, puérpera, mãe – como unidade indivisível. Para além do ciclo gravídico-puerperal, a mulher precisa ser apreendida em suas vivências e experiências singulares e indivisíveis.

A apreensão ampliada e contextualizada de assistência/cuidado, como unidade complexa – singular e multidimensional – é imprescindível para a indução da autonomia, da criatividade, da interatividade, enfim das relações próximas, dialógicas e humanizadas no contexto da saúde materno-infantil.

A presente pesquisa ressalta a importância e recomenda novos estudos acerca da necessária evolução de pensamento na assistência às gestantes e puérperas, a fim de promover abordagens integrativas e colaborativas entre os profissionais e destes com os usuários de saúde.
